# Square bananas, blue horses: the relative weight of shape and color in concept recognition and representation

**DOI:** 10.3389/fpsyg.2015.01542

**Published:** 2015-10-08

**Authors:** Claudia Scorolli, Anna M. Borghi

**Affiliations:** ^1^Department of Psychology, University of BolognaBologna, Italy; ^2^Institute of Cognitive Science and Technology, Consiglio Nazionale delle RicercheRome, Italy

**Keywords:** shape, color, animate self-moving entities, inanimate manipulable entities, categorization, manipulation judgments, motion judgments, biofunctional understanding

## Abstract

The present study investigates the role that shape and color play in the representation of animate (i.e., animals) and inanimate manipulable entities (i.e., fruits), and how the importance of these features is modulated by different tasks. Across three experiments participants were shown either images of entities (e.g., a sheep or a pineapple) or images of the same entities modified in color (e.g., a blue pineapple) or in shape (e.g., an elongated pineapple). In Experiment 1 we asked participants to categorize the entities as fruit or animal. Results showed that with animals color does not matter, while shape modifications determined a deterioration of the performance – stronger for fruit than for animals. To better understand our findings, in Experiments 2 we asked participants to judge if entities were graspable (manipulation evaluation task). Participants were faster with manipulable entities (fruit) than with animals; moreover alterations in shape affected the response latencies more for animals than for fruit. In Experiment 3 (motion evaluation task), we replicated the disadvantage for shape-altered animals, while with fruits shape and color modifications produced no effect. By contrasting shape- and color- alterations the present findings provide information on shape/color relative weight, suggesting that the action based property of shape is more crucial than color for fruit categorization, while with animals it is critical for both manipulation and motion tasks. This contextual dependency is further revealed by explicit judgments on similarity – between the altered entities and the prototypical ones – provided after the different tasks. These results extend current literature on affordances and biofunctionally embodied understanding, revealing the relative robustness of biofunctional activity compared to intellectual one.

## Introduction

The ability to recognize and to categorize objects and entities is crucial for our species. In order to survive, we need to recognize objects and entities and to decide whether to assign them to a given category or not. In the present work we will investigate with different tasks the role that shape and color play in the representation of animals and fruit. Specifically, we will deal with cases in which the exemplars that we see are quite different in shape and color from the typical members of the categories of animals and fruits. For example, we typically think of yellow bananas, and brown gorillas, so what if we see a blue, or a square banana? “Blue bananas, green gorillas," quotes a song for children (youtube.com/watch?v=kxSWGIm2XtI). If objects appear in a deformed color or shape, would we still recognize them? And, even if we recognize them, would we interact with them in an appropriate way? Finally, when directly contrasting color and shape, which one turns out to be more crucial for object recognition, categorization, and for interaction with objects?

The role of *shape* for object recognition has been underlined in a variety of theories. According to one of the most influential and sophisticated theories (Biederman, 1987; Biederman and Bar, 1999) objects are represented in terms of structural descriptions – specifying object parts and their relationships. In this theory, object recognition involves recognizing geometrical ions, geons, corresponding to object parts. The higher the number of parts, the more difficult recognition is. Disrupting these parts and their relations significantly impairs object recognition.

At a theoretical level, the strict interrelation between shape and action is particularly relevant for embodied and grounded (EG) theories of cognition (Barsalou, 2008; Borghi and Pecher, 2012; Borghi and Caruana, 2015). These views suggest that our understanding of the world is grounded in action, and that our body shapes the way we interact with objects and entities around us and the way we represent them.

### Shape and Action

Evidence from EG view have recently underlined the role of shape on object categorization, basically due to the relationship of shape with action (see Morlino et al., 2015). Well-known evidence on the so-called “shape bias” (e.g., Landau et al., 1988, 1992) has shown that, at least in Western societies, novel nouns are typically extended on the basis of shape similarity, rather than on the basis of similarity of color or texture. This bias is present in 2-year-olds and becomes rather stable from 3 years on. Smith (2005) has demonstrated that shape categories are not static and pre-defined, but that their formation is influenced by action: moving an object horizontally (or vertically) and moving it symmetrically (or asymmetrically) changes the range of shapes seen as similar. Using both a similarity evaluation task and an action-based sorting task, Iachini et al. (2008) compared novel objects varying in properties with a different degree of interactivity: grip (handle, broken handle), shape (triangle, square), size (large, small) and color (red, blue). The role of shape was clearly linked to action: shape played a role in the sorting task when participants were required either to observe and interact with the objects (Vision + Action) or to interact with them when blindfolded (Action). Color, instead, was not relevant across the two tasks.

Actually the importance of shape – due to structural reasons – is compatible with both EG and non-embodied views of cognition. The relationship between shape and action argues instead in favor of EG theories.

### Shape and Flexibility

Embodied and grounded theories have emphasized that affordances of objects are strictly related to objects’ shape. For some years studies on affordances – intended as opportunities to act that the environment offers to organisms (Gibson, 1979) – were mainly aimed at showing that affordances are activated in an automatic way, i.e., independently from the task at hand (e.g., shape, Tucker and Ellis, 1998). Nevertheless recent research has started to emphasize the flexibility rather than the automaticity in affordance activation. Evidence has shown that the activation of object affordances is modulated by the physical context (e.g., Yoon et al., 2010; Borghi et al., 2012; Kalenine et al., 2014), by the social context (e.g., Ellis et al., 2013; Gianelli et al., 2013; Scorolli et al., 2012, 2014), and by the task (Tipper et al., 2006; Pellicano et al., 2010; Cho and Proctor, 2011, 2013; Hsu et al., 2011).

Overall this evidence suggests that the role of shape in object recognition is affected by both the stimulus and the task at hand. This flexibility is compatible with EG views of cognition.

### Color and Object Recognition

While shape is typically included in all models of object recognition, the role of *color* is more disputed: it is definitely our aim to add a piece to this puzzle. Recent behavioral and brain imaging evidence has highlighted the importance of color for object recognition and conceptual representation (e.g., Martin et al., 1995; Chao and Martin, 1999; Kellenbach et al., 2001; Thompson-Schill, 2003; Connell and Lynnot, 2009; van Dam et al., 2012; Yee et al., 2012). A recent meta-analysis on 35 experiments (Bramão et al., 2011) confirmed that color improves object recognition, mostly for color diagnostic objects but also for non- color diagnostic ones. Color facilitated object recognition mainly in naming tasks, while it had only a marginally significant effect in semantic categorization tasks and it didn’t play a significant role in property verification task. As to property verification task, findings are not straightforward: using a naming and a property verification task, Therriault et al. (2009) found that color information is regularly used in recognition, at least when diagnostic colors are concerned. In a similar vein, Simmons et al. (2007) required participants to perform a property verification task indicating whether a color or a motor property was typically true of a given object (e.g., TAXI-yellow, HAIR-combed); then they asked participants to perform a color perception task. Through fMRI they found that a region of the left fusiform gyrus was active both during color perception and during access to conceptual knowledge on color.

Further studies have investigated the role of color with different kinds of entities showing that color is more relevant for categorization of natural objects compared to artifacts. For example, Price and Humphreys (1989) found that naming of natural objects was facilitated by color. This can be due to the fact that their members are more similar, and color information serves to solve the competition between different members; an alternative explanation ascribes this difference to the fact that color is typically more diagnostic for natural objects.

Overall the above reviewed literature provides evidence of a strong impact of shape on object recognition; this impact is strongly modulated by the stimulus and the task at hand. However, also color information seems to play an important role: it should definitively be included in models of object recognition, particularly for color-diagnostic objects as natural objects. Nevertheless the small amount of empirical studies does not allow to build a model addressing also the issue of color-information flexibility. As to relevance for EG views, it is evident that color is not structurally linked to action: its importance can be still compatible with both EG and non-embodied views of cognition. Nevertheless its eventual flexibility would argue in favor of EG theories, according to which the way objects afford actions and the way knowledge on object categories is accessed is contextually modulated and flexible.

### Open Issues

With the present study we intend to address the open issues left from the overviewed literature:

(a) Non-embodied research on object recognition has typically considered *shape* as a static object property. This literature has neglected the relationship between objects and dynamic bodily actions. In the vein of EG perspectives, we intend to further address the idea that the relevance of shape for object recognition is not just due to structural reasons but to its importance for action.
(b) In the majority of the studies – from both embodied and non-embodied literature – *color* was considered *per se* or compared with other surface properties, such as texture, and only rarely with shape. Furthermore, even if differences in the role played by color with natural objects and artifacts have been investigated, more fine-grained differences within natural objects – and the possible modulation determined by the task at hand – have not been fully explored.

To address these issues we decided to directly contrast shape and color, in order to disambiguate for *which tasks* shape is more/less relevant than color, as well as *why* they can play different roles.

To this aim in the present work we adopted the method suggested by the song: participants were presented with figures of common animals and fruits, with standard or deformed color and shape (e.g., yellow oblong bananas – baseline – vs. yellow square bananas vs. blue oblong bananas) and were asked to respond by pressing two different keys on the keyboard (see Bazzarin et al., 2007, for a similar method used to investigate the effects of variations in object size). We tested how and to what extent deformations in color and shape differently influence the category assigned to the items (Experiment 1) as well as judgments on manipulability or on motion (Experiments 2 and 3).

From a theoretical point of view our decision was to select entities with which we typically act or not (manipulable vs. non manipulable objects) and to use tasks that put a different emphasis on categorization (animals vs. fruits: Experiment 1), on action (manipulable vs. non-manipulable objects/entities: Experiment 2) or on motion (animate and self-moving vs. non-animate objects/entities: Experiment 3). We decided to focus on natural objects as they are more color-diagnostic than artifacts. Within natural objects we selected manipulable inanimate objects which are typically acted upon (i.e., fruits and vegetables) and non-manipulable and animate entities endowed with self-propelled motion (i.e., animals). We altered in turn their color or their shape (for studies on manipulable objects, see Gerlach et al., 2002; Boronat et al., 2005; Borghi et al., 2007; Marino et al., 2011a,b; Rueschemeyer et al., 2011).

The overall work is structured in the following way: after Experiment 1, in which a standard categorization task was employed, Experiment 2 was designed to investigate if participants would perform similarly to the categorization task also in a different task, that directly triggers information on manipulation. Then a motion judgment task (Experiment 3) was employed to test the effects of color and shape in a task that does not refer at all to potential interactions. Through independent ratings we also investigated if the previous performed task (i.e., categorization vs. manipulation vs. motion tasks) modulates explicit judgments on the same entities.

In line with both the literature and the research directions suggested by the EG theories of cognition we advance the general prediction that shape will have a prominent role across the tasks and the entities. The first reason underlying the importance of shape is that altering the shape of an object/entity provokes a modification of its structure. More crucially to us, another reason might underlie the relative importance of shape: while color is only a visual property, shape processing involves both the visual and the tactile/motor system, and it is directly related to action (Smith, 2005). However, we predict that also color does have a weight, specifically that the relative weight of shape and color strongly depends on both the specific entity and the task at hand.

Resting on EG views, and specifically on a view where biofunctional understanding underlies psychological comprehension (Iran-Nejad, 2011), we formulate the following predictions:

(1) The three kinds of tasks should have different effects on stimuli processing – not explained by explicit judgments – since objects are conceived in terms of the possible actions we can perform on them (categorization task), but the specific task at hand could trigger a more fine-grained action (manipulation evaluation task) and/or force to focus on the entity self-propelled movement, that is rather independent from the observer (motion evaluation task).

In particular:

(2) Fruits: if objects are represented as patterns of potential action, rather than simply in terms of their perceptual characteristics (e.g., Wilson, 2002; Borghi and Pecher, 2012; Borghi and Caruana, 2015), with both a categorization and a manipulative task the property of shape should be crucial. This prediction is derived from the EG cognition views, according to which entities are conceived in terms of the possible actions we can perform with them. Nevertheless – if cognition is not only ‘based on’ but also ‘oriented to’ action – with a task directly triggering manipulation, color should affect performance similarly to shape, as this task does activate a precise simulation of the agent-object interaction. In this case, the color of a fruit could be diagnostic of its eventual toxicity, or of its degree of ripening (e.g., a rotten apple). On the contrary, for a motion evaluation task neither the alterations of shape nor of color should affect the performance with inanimate – non-self-moving entities.
(3) FAnimals: shape should be more important for a manipulation evaluation task than for a categorization task, as the first forces us to focus on a possible manipulation of the entity, that we are less used to directly handle compared to fruits. Shape should have also a stronger weight than color with a motion evaluation task: altered shape, as well as missing parts, would severely modify self-moving entities’ movement, and/or influence their status of living beings. The prediction that structural shape modifications would be particularly effective for animals in the motion evaluation task is derived from an embodied cognition view, since structural changes would drastically modify their capability to move. Actually a structural role of shape could be predicted also by disembodied theories of cognition, but these theories would predict a strong decay of participants’ performance for shape alterations *regardless of* the kind of task (that is, also for the categorization task).
(4) FFinally, if cognition is based on our previous sensory-motor experience with the external world, the previously executed task should determine also a long lasting effect on the perceived similarity between the manipulated entities and the prototypical ones. We still expect a powerful effect of shape alterations, but – more relevant to our hypotheses – we predict that participants who have previously performed a manipulation task will judge the shown entities as more similar to the imagined-prototypical ones compared to both participants who hadn’t previously performed any task and participants who had previously performed a motion evaluation task.

## Semantic Categorization Task

Experiment 1 was designed to test the role played by color and shape in a standard categorization task. We focused on natural objects – since they are typically considered as color-diagnostic entities – and contrasted manipulable and non-manipulable entities.

### Participants

Twenty students from the University of Bologna took part in the experiment (10/20 were females). All were native Italian speakers, right-handed, and had normal or corrected-to- normal vision; they gave their informed consent to the experimental procedure. Their ages ranged from 19 to 30 years old (*M* = 23.40; *SD* = 3.19). The study was approved by the local ethic committees (Department of Psychology, University of Bologna).

### Materials

**FIGURE 1:**
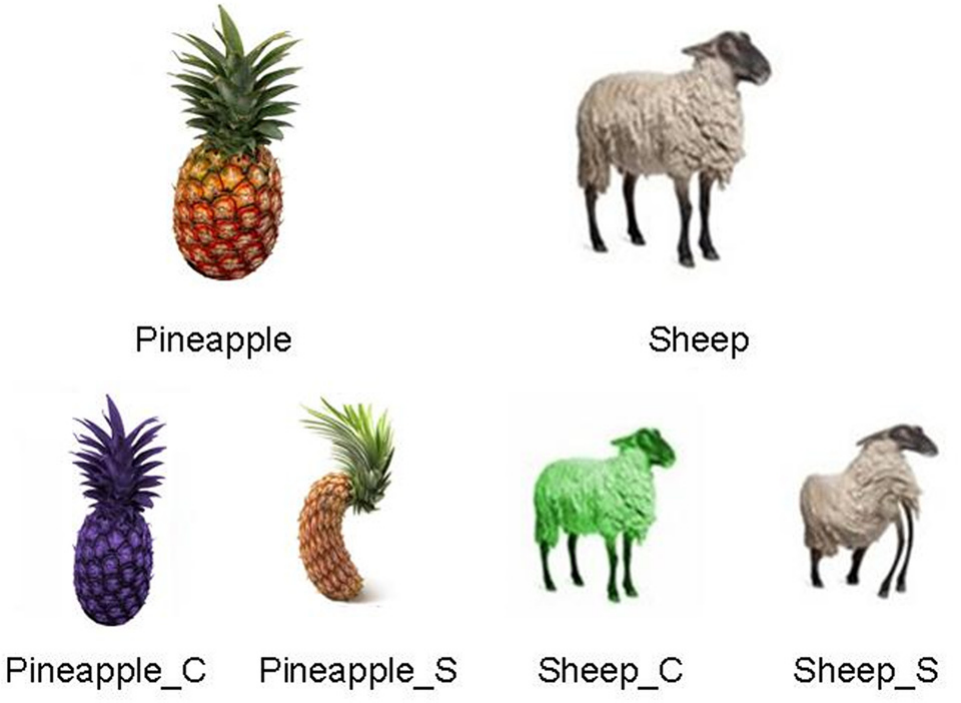
**(Above)** Pictures of a pineapple (manipulable, not self-moving entity) and a sheep (non-manipulable, self-moving entity); **(below)** the pictures of both the fruit and the animal were altered in color (_C) or in shape (_S).

We selected 16 pictures of fruits and vegetables (pineapple, orange, apricot, avocado, banana, carrot, cherry, strawberry, kiwi, almond, apple, blackberry, walnut, hot pepper, peach, grapes) and 16 of animals (dog, rabbit, cat, red snapper, shark, turtle, parrot, mouse, rooster, pig, horse, lion, giraffe, elephant, crocodile, sheep), having a typical color and shape (baseline). Then we modified these pictures, to obtain 64 further pictures of fruits and animals, altered in turn in shape or color (see two examples in **Figure 1**). Overall the stimuli consisted of 96 images.

As far as materials assessment is concerned, first of all it is worth noting that image manipulation software products (e.g., GIMP 2, the open-source raster graphics editor we used) do not allow fixing shape and color modifications to the same degree of distortions for both the properties. Secondly, even if in literature we did not find paradigms similar to ours, for sure volumetric properties typically affect objects recognition more than surface properties (Banno and Saiki, 2015; Reppa et al., 2015; Rosselli et al., 2015; Saarela and Landy, 2015; Schlangen and Barenholtz, 2015). However, as far as our specific hypotheses were concerned, it was crucial to ascertain that the perceptual complexity of both the color- and the shape-modified stimuli was comparable across stimuli (fruits-vegetables and animals). In order to check if ‘our’ color and shape distortions could be comparable across the different domains (fruits-vegetables vs. animals) an independent group of twelve participants (seven females, ages ranging from 26 to 32 years old, *M* = 28.08, *SD* = 2.23) was presented with each entity in its original shape and color and – immediately below – with the same entity altered for shape or color (both the stimuli were simultaneously visible in the computer screen). Each participant was asked to rate the degree of similarity between the two entities (“identical” – “completely different”) on a graduated 100-point scale. The collected scores underwent a 2 (Stimulus: Animal vs. Fruit) × 3 (Alteration: Color vs. Shape vs. Absent) mixed factor ANOVA, with both variables manipulated within participants. The ANOVA showed a main effect of alteration, *F*(2,90) = 456.44, *MSE* = 49.21, *p* < 0.01: as expected, shape modifications altered the entities more than color modifications (*M* = 65.40, *SD* = 7.56; *M* = 42.16, *SD* = 7.94), *post hoc* Tukey’s Honestly Significant Difference (HSD): *p* < 0.01. Nevertheless we did not found differences between fruits and animals, nor an interaction between the kind of entity and the kind of alteration. Finally we performed separate analyses for animals and fruits: we found basically the same pattern for both entities, that is a significant effect of the kind of alteration due to stronger dissimilarities judgments provided for shape-alterations than for color-alterations (*post hoc* Tukey’s HSD: *p*s < 0.01: Animals: shape-alterations = 68.89; color-alterations = 42.04; baseline = 12.64; Fruits: shape-alterations = 62.92; color-alterations = 42.27; baseline = 12.45). Therefore, even if explicit judgments provided by the independent group of participants showed greater perceptual complexity of shape-modified stimuli compared to color-modified ones, crucially color and shape alterations affected fruits and animals in a similar way. As we found no differences in similarity judgments across different stimuli, reasonably altered fruits and animals should be comparable in the experimental tasks.

To further check if the chosen distortions allow observers to categorize the entities as fruits-vegetables or animals, an independent group of ten participants (eight females, ages ranging from 28 to 42 years old, *M* = 32.00, *SD* = 5.76) was asked (1) to decide whether each depicted entity – natural entity, color-modified entity, shape-modified entity – was an animal or a fruit-vegetable; (2) to specify its name (as to context effects in naming see Malt, 2013). The stimuli were presented on a computer screen; the answers were collected by the experimenter. Two random orders of the stimuli were used; the original version of the entities (i.e., not modified either in color and shape) followed the altered versions. Crucially, all participants correctly distinguished animals from fruits-vegetables; moreover they rightly named all the entities except the shape-modified turtle and the shape-modified crocodile (7/10 and 6/10 participants, respectively, were not able to correctly name the entities, nevertheless they identified them as animals), the shape-modified cherry (2/10 participants produced the name ‘red pepper,’ i.e., still a vegetable) and the orange (it was categorized as ‘lemon’ by two persons, i.e., still a fruit).

As none of our tasks required to correctly identify the individual entity, but only to categorize them as fruits-vegetables vs. animals, or as manipulable non-self-moving entities vs. non-manipulable/self moving entities, we used the selected entities across all the three experiments.

### Procedure

Participants were tested individually in a quiet laboratory room. They sat on a comfortable chair in front of a computer screen and were instructed to look at a fixation cross (+) that remained on the screen for 500 ms. When the fixation cross disappeared, a photograph of one object (6° × 6°visual angle degree) appeared on the screen for 1000 ms. The timer started operating when the image appeared on the screen. All stimuli were displayed centrally on the monitor and randomized. The experiment was programmed using E-prime2 software (Psychology Software Tools). Each object was seen once by each participant.

For each image, half of the participants were instructed to press one key (‘M,’ on the right) with the right hand if the shown object was an animal; they had to press a different key (‘X,’ on the left) with the left hand if the object was a fruit-vegetable. The other half of the participants performed the same task with the opposite hand mapping. Participants received feedback for both correct and incorrect responses. All participants were informed that their response times would be recorded and were asked to respond as quickly as possible while still maintaining accuracy. The 96 experimental trials were preceded by six practice trials (a lemon and a hedgehog: natural colored and shaped/altered for color and shape), in order to allow participants to familiarize themselves with the procedure.

### Statistical Analysis

The percentage of errors underwent a 2 (Mapping: Right hand–Fruit and Left hand– Animals vs. Left hand–Fruit and Right hand–Animals) × 2 (Stimulus: Animal vs. Fruit) × 3 (Alteration: Color vs. Shape vs. Baseline) mixed factor ANOVA, with Mapping as between- participants variables. We conducted the analyses with participants as a random factor. After eliminating all incorrect responses, RTs were submitted to an ANOVA with the same factors. Finally we performed two separate analyses for animals and fruit: RTs were submitted to a 2 (Mapping) × 2 (Alteration) mixed factor ANOVA, with Mapping as between-participants variable. We used the Tukey’s HSD procedure to correct for multiple means comparisons as it affords the same protection as the Bonferroni adjustment when comparing many treatment groups, but it also makes easier to reject the hypothesis of no difference when there are real differences between the groups. We did not perform a prospective power analysis, but only a retrospective one (Hoenig and Heisey, 2001).

### Results

#### Overall

As to percentage of errors, we found no significant effects. Analyses on RTs showed a main effect of the kind of alteration, *F*(2,36) = 7.23, *MSE* = 12684, $\eta_{p}^{2}$ = 0.29, *observed power* = 0.91, *p* < 0.002, as participants responded significantly slower to odd shaped stimuli (*M* = 558 ms) than to baseline (*M* = 523 ms, *post hoc* Tukey’s HSD: *p* < 0.002). Odd shaped stimuli slightly differed from odd colored stimuli (*M* = 537 ms, *post hoc* Tukey’s HSD: *p* = 0.07). The baseline did not differ from the color-modified condition.

#### Animals

**Percentage of errors**. *M* = 1.06%; *SD* = 1.87. We found no significant effects.

**RTs.** The ANOVA showed a main effect of alteration, *F*(2,36) = 4.68, *MSE* = 6520, *p* < 0.02: participants responded slower to odd shaped stimuli (*M* = 554 ms) than to baseline (*M* = 519 ms), *post hoc* Tukey’s HSD: *p* < 0.01 (see **Table 1**). There was no significant difference between the color- and the shape-alterations conditions (*p* = 0.63).

**Table 1:** The table summarizes the Response Latencies from the three Experiments (i.e. categorization; manipulation; agency).

	Experiment 1		Experiment 2		Experiment 3	
	Categorization		Manipulation		Motion	
Alteration	*A*	*F*	*A*	*F*	*A*	*F*
Color	544 ms (87)	530 ms (72)	479 ms (70)	471 ms (83)	557 ms (131)	558 ms (127)
Shape	554 ms (110)	562 ms (102)	517 ms (90)	493 ms (83)	589 ms (158)	573 ms (113)
Absent (baseline)	519 ms (79)	526 ms (74)	465 ms (67)	458 ms (69)	557 ms (139)	551 ms (133)

*Standard deviations are shown in parentheses*.

#### Fruit

**Percentage of errors**. *M* = 1.18%; *SD* = 1.43. We found no significant effects.

**RTs.** Analyses with fruit produced a main effect of alteration, *F*(2,36) = 5.01, *MSE* = 7562, *p* < 0.02: participants responded slower to odd shaped stimuli (*M* = 562 ms) than to baseline (*M* = 526 ms) and to odd colored stimuli (*M* = 530 ms), *post hoc* Tukey’s HSD: *p*s < 0.04 (see **Table 1**). The color modified condition did not differ from the baseline one (*p* = 0.93).

### Discussion

The results show that with a task directly activating categorical knowledge, with both manipulable (i.e., fruit) and non-manipulable entities (i.e., animals) color does not matter, while shape modifications determine a deterioration of the performance. Interestingly this deterioration was stronger for fruit (odd-shaped stimuli differed from both the baseline and the odd-colored condition) than for animals (odd-shaped stimuli did not differ from the odd-colored condition).

Crucially these findings cannot be explained resting on the selected stimuli, as in the materials assessment (see above) we found that shape-alterations affected explicit judgments on the two kinds of entities (fruits and animals) in a similar way; the same was true for color- alterations.

## Task Dependency: Manipulation and Motion Evaluation Tasks

### Manipulation

Experiment 2 was designed to investigate if participants would perform similarly to the categorization task in a task that directly involves access to object manipulability. Participants were asked to judge if they could grasp and lift with their right-hand the displayed entities to put them inside a black backpack located in front of the them (for a similar task see Borghi et al., 2007; Hirose et al., 2010).

#### Method

Nineteen students from the University of Bologna took part in the experiment (10/19 were females). All were native Italian speakers and right-handed by self-report; all had normal or corrected-to-normal vision. They all gave their informed consent to the experimental procedure. Their ages ranged from 20 to 30 years old (*M* = 23.50; *SD* = 2.96). The study was approved by the local ethic committees (Department of Psychology, University of Bologna).

We used the same materials and procedure as in Experiment 1, except for the task: for each image, 10 participants were instructed to press one key with the right hand if the shown object could be lifted with a hand and put inside a backpack. The backpack was located in front of them, on the right side of the computer display. If the object could not be lifted and put inside the backpack they had to press a different key with the left hand. Nine participants performed the same task with the opposite hand mapping. After the experiment, participants rated how each depicted object differed from how they would imagine it (see Long Term Effect of Task: Explicit Judgments).

The percentage of errors underwent a 2 (Mapping: Right hand–yes and Left hand–no vs. Right hand–no Left hand–yes) × 2 (Stimulus: Animal vs. Fruit) × 3 (Alteration: Color vs. Shape vs. Baseline) mixed factor ANOVA, with Mapping as between-participants variables. We conducted the analyses with participants as a random factor. After eliminating all incorrect responses, RTs were submitted to an ANOVA with the same factors. Finally we performed two separate analyses for animals and fruit: RTs were submitted to a 2 (Mapping) × 2 (Alteration) mixed factor ANOVA, with Mapping as between-participants variable.

#### Results

##### Overall

As to percentage of errors, we found a main effect of the kind of mapping: with mapping “Right hand–yes, the entity can be put inside the backpack” participants produced significantly more errors (*M* = 2.80%) than with the opposite mapping (*M* = 0.77%): *F*(1,17) = 7.73, *MSE* = 116.37, *p* < 0.02. We found also an interaction between the kind of mapping and the kind of stimulus: *F*(1,17) = 5.56, *MSE* = 9.71, *p* < 0.04, basically due to the fact that both with animals and fruits participants produced more errors with the mapping “Right-hand yes” (A: *M* = 3.26%; F: *M* = 2.33%) than with the opposite mapping (A: *M* = 0.66%; F: *M* = 0.88%), *post hoc* Tukey’s HSD: *p*s < 0.01.

Analyzing RTs we found a main effect of the kind of stimulus *F*(1,17) = 5.06, *MSE* = 4310.21, $\eta_{p}^{2}$ = 0.23, *observed power* = 0.56, *p* < 0.04 as participants were faster with fruits (*M* = 474 ms) than with animals (*M* = 486 ms). Analyses showed also a main effect of the kind of alteration: *F*(2,34) = 8.51, *MSE* = 18564, $\eta_{p}^{2}$ = 0.33, *observed power* = 0.95, *p* < 0.001: *post hoc* Tukey’s HSD showed that shape- alterations (*M* = 504 ms) differed from the baseline condition (*M* = 461 ms) and deteriorated the performance more than color-alterations (*M* = 475 ms, *p*s < 0.03).

##### Animals

**Percentage of errors.** *M* = 2.03%; *SD* = 2.41. We found a main effect of Mapping, as participants performed better when they had to respond to non-manipulable objects (animals) with the right hand (*M* = 0.66%) than with the left one (*M* = 3.26%), *F*(1,17) = 9.64, *MSE* = 9.24, *p* < 0.01.

**RTs.** The ANOVA on RTs showed a main effect of alteration, *F*(2,34) = 9.41, *MSE* = 13244, *p* < 0.001: participants responded slower to odd shaped stimuli (*M* = 517 ms) than to baseline (*M* = 465 ms) and to odd colored stimuli (*M* = 479 ms), *post hoc* Tukey’s HSD: *p*s < 0.01. The baseline condition did not differ from the color alteration condition (*p* = 0.22; see **Table 1**).

##### Fruits

**Percentage of errors.** *M* = 1.64%; *SD* = 1.83. With fruits we found a trend effect of Mapping, as participants performed slightly better when they had to respond to fruits with the left hand (*M* = 0.89%) than with the right one (*M* = 2.33%), *p* = 0.053.

**RTs.** As with animals, analyses on fruits showed a main effect of alteration: *F*(2,34) = 3.99, *MSE* = 6047, *p* < 0.03: odd shaped fruits differed only from the baseline (*post hoc* Tukey’s HSD: *p* < 0.02) and not from the odd colored ones. The baseline condition did not differ from the color alteration one (*post hoc* Tukey’s HSD*: p* = 0.57; see **Table 1**).

#### Discussion

Results from this experiment show an effect of mapping on participants’ accuracy. When the participants had to respond with the dominant hand for “yes, the entity can be put inside the backpack,” they produced more errors than for the symmetric kind of response (left hand), likely due to an interference effect. As the effect is significant only for animals, and not for fruit, it could be due to the unfeasibility of action in case of non-manipulable entities.

As to analyses on RTs, interestingly we found that participants were faster with fruits than with animals. Looking at the separate analyses, shape alteration deteriorated the performance more with animals than with fruits (as for animals the odd-shaped stimuli differed from both the baseline and the odd-colored condition). This result differs from our prediction of a similar effect of shape and color alterations on fruit in case of a manipulation task. This finding could be due to the fact that the manipulative task forced participants to focus on a possible manipulation of entities that they are less used to directly handle (animals) compared to fruits.

### Motion

In the last Experiment we tested the same stimuli, but using a task that triggers information on agency and self-propelled motion (see Newell et al., 2004). We expect an effect of shape alteration affecting not fruits (differently from Experiments 1 and 2), but animate entities, since shape modifications should render animals’ self-propelled motion more difficult.

#### Method

An independent group of nineteen students from the University of Bologna took part in the third experiment (11 females). All were native Italian speakers, right-handed by self- report; all had normal or corrected-to-normal vision. They all gave their informed consent to the experimental procedure. Their ages ranged from 19 to 38 years old (*M* = 24.75; *SD* = 6.43). The study was approved by the local ethic committees (Department of Psychology, University of Bologna). We used the same materials and procedure of previous experiments, except for the task, as for each image 10 participants were instructed to press one key with the right hand if the shown object typically moves; they had to press a different key with the left hand if the object cannot move on its own. Nine of the participants performed the same task with the opposite hand mapping. As in Experiment 2, after this experiment, participants rated how each depicted object differed from how they would imagine it (see Long Term Effect of Task: Explicit Judgments).

Similarly to the previous experiment, we performed an overall analyses and then two separate analyses for animals and fruits.

#### Results

##### Overall

We found no effect of the percentage of errors. We found a main effect of the kind of alteration on response times: *F*(2,34) = 6.66, *MSE* = 7845, $\eta_{p}^{2}$ = 0.28, *observed power* = 0.89, *p* < 0.005: *post hoc* Tukey’s HSD showed that shape-alterations (*M* = 580 ms) differed from the baseline condition (*M* = 554 ms) and deteriorated the performance more than color-alterations (*M* = 557 ms, *p*s < 0.02).

##### Animals

**Percentage of errors.** *M* = 2.07%; *SD* = 2.61. No significant effect was found.

**RTs.** Analyses showed a main effect of alteration, *F*(2,34) = 7.44, *MSE* = 6548, *p* < 0.003: participants responded slower to odd shaped stimuli (*M* = 589 ms) than to baseline (*M* = 557 ms) and to odd colored stimuli (*M* = 557 ms), *post hoc* Tukey’s HSD: *p*s < 0.007. The baseline condition did not differ from the altered-color condition (*post hoc* Tukey’s HSD *p* = 0.99, see **Table 1**).

##### Fruits

**Percentage of errors**. *M* = 2.21%; *SD* = 3.12. As for animals, no significant effect was found.

**RTs.** Analyses on RTs showed no significant effect with fruits (*p* = 0.21, see **Table 1**).

#### Discussion

Response latencies revealed a strong effect of shape alterations with animals, while color modifications had no effect. This is likely due to the fact that animals are characterized by self-motion, and that shape disruptions might significantly hinder their movement (for an investigation also on the feature of orientation on moving objects see Jardine and Seiffert, 2011). Given that no biological movement (e.g., Jastorff and Orba, 2009) nor apparent motion (see Scholl and Tremoulet, 2000) was displayed, judgments on motion for animals are strongly affected by shape. With fruit no effect of shape (nor of color) was present. This could be due to the fact that fruits are not characterized by self-motion but by actions one can perform on them (Setti et al., 2009): they could be easily categorized as stationary.

### Possible Effects of Scaling Across Entities

As the size of all the depicted entities was the same – across both kinds of stimuli – to assess for possible effects of scaling an independent group of 10 participants (eight females, ages ranging from 28 to 42 years old, *M* = 32.00, *SD* = 5.76) was presented with the entities in their original shape and color and were asked to rate their actual size, regardless of the image’s size, using a four-point scale: “very small,” “small,” “large,” “very large.” The stimuli were presented on a computer screen; the answers were provided by marking the corresponding box. Two random orders of the stimuli were used. As a reference frame we showed participants four new entities, which were neither animals nor fruits: a marble (very small), a mug (small), a backpack (large) and a chest (very large). As to animals, horse, shark, lion, giraffe, crocodile, elephant, were consistently categorized as ‘very large’; dog, rabbit, cat, pig, sheep, parrot, cock as ‘large’; red snapper, mouse as ‘small’; turtle was judged as small by 7 out of 10 participants. As to fruits, pineapple, orange, avocado, banana, carrot, kiwi, apple, apricot, grapes were categorized as ‘small’; cherry, strawberry, almond, blackberry, walnut, hot pepper as ‘very small.’ This assessment allowed us to introduce the overall four-levels factor of Scaling: very large scale (for “very small” entities), large scale (for “small” entities), small scale (for “large” entities), and very small scale (for “very large” entities). As none of animals was categorized as “very small,” for animals the levels of Scaling were: Large, Small and Very Small. Symmetrically, as none of fruits-vegetables was categorized as “large” or “very large,” for fruits-vegetables the levels of Scaling were: Very Large and Large.

For all the three experiments, we investigated the possible effect of different kinds of scaling by performing two separate analyses for animals and fruits, with *items* as a random factor. For animals RTs were submitted to a 3 (Alteration: Color vs. Shape vs. Absent, baseline) × 3 (Scaling: Large scale vs. Small scale vs. Very Small scale) mixed factor ANOVA; for fruits RTs were submitted to a 3 (Alteration: Color vs. Shape vs. Absent, baseline) × 2 (Scaling: Very Large scale vs. Large scale) mixed factor ANOVA. Crucially, the effect of scaling was consistent and not significant across the three experiments (*p*s < 0.70). For conciseness we will show analyses only for the *Categorization task*: with animals, ANOVA on items only showed a main effect of the alteration [*F*(2,12) = 4.17, *MSE* = 5361.92, *p* < 0.05], but not an effect of the Scaling, *p* = 0.36. Similarly, ANOVA on fruits showed a main effect of alteration [*F*(2,13) = 3.52, *MSE* = 12.37.22, *p* < 0.05] and no effect of the Scaling, *p* = 0.85.

## Long Term Effects of the Tasks: Explicit Judgments

To evaluate whether the specific kind of task determined also a long lasting effect on the evaluation on the similarity between the shown objects and the imagined ones, at the end of the manipulation and motion judgments tasks, participants rated how each depicted object differed from how they would imagine it. The obtained scores were compared to the scores collected from an independent group of twenty participants (11 females, ages ranging from 25 to 40 years old; *M* = 32.00; *SD* = 4.23) who did not perform any previous task.

### Method

All three groups of participants were informed that during the rating three versions of the same entity were shown: standard, modified in shape, modified in color. The stimuli were presented on a computer screen; two random orders of the stimuli were used; each item was shown once. Participants were asked to rate how each depicted object (both the standard and the altered ones) differed from their own prototypical representation of that entity, i.e., from how they would imagine it (“very similar” – “not similar” at all. For an analogous assessment see also the Verbalizer–Visualizer Questionnaire by Kirby et al., 1988). We used a discrete interval scale, with scores ranging from 0 to 100. To familiarize participants with the procedure, at the beginning they were asked to perform the same rating with two entities, in the standard and modified (color/shape) conditions; during this phase they could ask for explanations to the experimenter. We calculated the scores’ averages for each condition.

As the distribution of data was found to be not significantly different from the normal one (Shapiro–Wilk test), the scores were submitted to a 3 (Alteration: Color vs. Shape vs. Absent – baseline) × 3 (previous Task: absent vs. manipulability vs. agency) mixed factor ANOVA, with previous Task as between-participants variable. We conducted the analyses with participants as a random factor. If objects are represented as patterns of potential actions, then participants should perceive the shown entities as more similar to their prototypical representations after a task focusing on manipulation (Borghi and Riggio, 2009); the effect should be particularly strong for manipulable entities, i.e., fruits.

### Results

#### Animals

We found a significant main effect of alteration, *F*(2,45) = 439.51, *MSE* = 150.39, *p* < 0.0001: participants rated “imagined” animals as more similar to baseline objects (*M* = 5.11) than to odd colored ones (*M* = 41.90, *post hoc* Tukey’s HSD, *p* < 0.0001), which in turn differed from odd shaped stimuli (*M* = 79.32, *p*s < 0.0001). Analyses also showed a main effect of the task, *F*(2,45) = 140.91, *MSE* = 12.46, *p* < 0.0001, as the shown objects were judged more similar to the imagined-prototypical ones after the manipulation evaluation task (*M* = 35.20) than after the motion evaluation task (*M* = 44.72, *post hoc* Tukey’s HSD, *p* < 0.05), which in turn differed from the baseline condition (*M* = 46.43, *p*s < 0.05).

**FIGURE 2:**
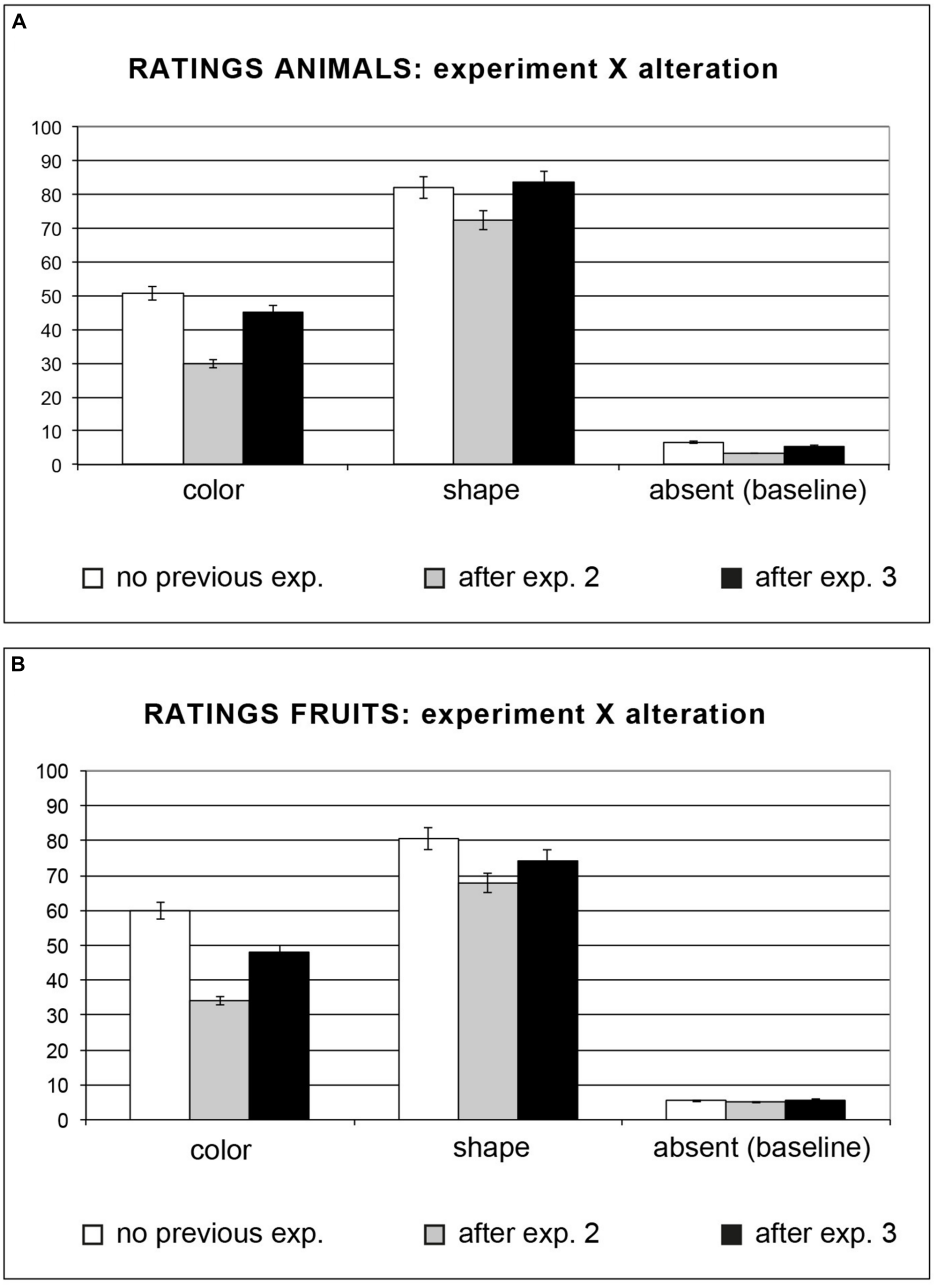
**(A,B)** Participants were asked to rate how each depicted object differed from how they would imagine them. Both with animals **(A)** and with fruits **(B)** we found an interaction between the experiment and the alteration. Error bars represent the standard error.

Interestingly, we found an interaction between the alteration and the task, *F*(2,90) = 30.13, *MSE* = 12.46, *p* < 0.0001: the participants’ scores for *odd colored* animals were significantly lower after the motion evaluation task (*M* = 45.16) compared to the baseline condition (*M* = 50.74, *post hoc* Tukey’s HSD, *p* < 0.001); after the manipulation evaluation task they judged the entities as very close to the imagined-prototypical ones (*M* = 29.82, *post hoc* Tukey’s HSD, *p*s < 0.001; see **Table 2**). The explicit judgments for *odd shaped* animals did not differ for ‘absence of a previous task’ (*M* = 81.98) and ‘previous experiment on motion’ (*M* = 83.61), while after the manipulability task the participants’ scores were lower (*M* = 72.40, i.e., “very similar”) compared to the other two conditions (*p*s < 0.001). Finally, scores for the *baseline condition* did not differ across conditions: task absent: *M* = 6.57; manipulability: *M* = 3.40; agency *M* = 5.38, *post hoc* Tukey’s HSD *p*s > 0.99; see **Table 2** and **Figure 2A**).

**Table 2:** The table summarizes the mean scores from the three Ratings (i.e., without previous task; after the manipulation task; after the motion evaluation task).

	Any previous		After manipulation		After motion	
	task		task		evaluation task	
Alteration	*A*	*F*	*A*	*F*	*A*	*F*
Color	50.74	59.79	29.82	34.08	45.16	47.96
	(14.08)	(12.21)	(11.13)	(9.01)	(8.14)	(6.86)
Shape	81.98	80.65	72.40	67.82	83.61	74.28
	(7.12)	(11.21)	(4.57)	(7.55)	(5.96)	(7.20)
Absent	6.57	5.38	3.40	5.09	5.38	5.71
(baseline)	(4.27)	(2.81)	(3.04)	(2.28)	(1.58)	(2.29)

*Standard deviations are shown in parentheses*.

#### Fruits

A significant main effect of alteration was present with fruit as well, *F*(2,45) = 378.18, *MSE* = 152.80, *p* < 0.0001: as for animals, participants rated “imagined” fruits as more similar to baseline objects (*M* = 5.40) than to odd colored ones (*M* = 47.28, *post hoc* Tukey’s HSD, *p* < 0.0001), which in turn differed from odd shaped stimuli (*M* = 74.25, *p*s < 0.0001). Analyses also showed a main effect of the task, *F*(2,45) = 168.06, *MSE* = 11.98, *p* < 0.0001: as found with animals, the shown fruits were judged more similar to the imagined-prototypical one after the manipulation evaluation task (*M* = 35.67) than after the motion evaluation task (*M* = 42.65, *post hoc* Tukey’s HSD, *p* < 0.0001), which in turn differed from the baseline condition (*M* = 48.60, *p*s < 0.001).

We found an interaction between the alteration and the task, *F*(2,90) = 54.06, *MSE* = 11.98, *p* < 0.0001: the participants’ scores for *odd colored stimuli* were significantly lower after the motion evaluation task (*M* = 47.96) compared to the baseline condition (*M* = 59.79, *post hoc* Tukey’s HSD, *p* < 0.0001); after the manipulation evaluation task they judged the shown entities as very similar to the imagined-prototypical ones (*M* = 34.08, *post hoc* Tukey’s HSD, *p*s < 0.001; see **Table 2** and **Figure 2B**). Unlike for animals, the performance for *odd shaped* fruits without previous task (*M* = 80.65) and after the motion evaluation task (*M* = 74.28) significantly differed (*post hoc* Tukey’s HSD *p* < 0.001), as the motion evaluation task seemed to render the shown fruits more similar to the imagined ones; as for *odd colored* fruits, after the manipulation evaluation task the participants obtained the lowest scores (*M* = 67.83, i.e., “very similar”, *p*s < 0.001). As for animals, scores for the *baseline condition* did not differ in the case of ‘no previous task ratings’ (*M* = 5.38), ‘previous manipulation evaluation task’ (*M* = 5.09) and ‘previous motion evaluation task’ (*M* = 5.71, *post hoc* Tukey’s HSD *p*s > 1; see **Table 2** and **Figure 2B**).

### Discussion

Analyses on explicit judgments on similarity showed that the match between the shown and the imagined-prototypical entities was rendered more difficult by the alteration of shape, rather than by the alteration of color, for both animals and fruit. In addition, participants who had previously performed a manipulation evaluation task (Experiment 2) judged shown animals and fruits as more similar to the imagined-prototypical entities compared both to participants who hadn’t previously performed any task and to participants who had previously performed a motion evaluation task (Experiment 3).

The first result on explicit judgments confirms the prominence of shape over color. The second result, consistent for both animals and fruit, indicates that objects are interpreted in terms of the potential actions we can perform on them (manipulation) rather than in terms of their own “independent” movement (agency, self-propelled motion; see Setti et al., 2009, for a similar conclusion). Consistent with this interpretation, analyses on scores showed that, after the manipulation judgment, entities altered both in shape and color were judged as more similar to the imagined-prototypical ones if compared to the same pictures without a previous task. Interestingly, also the motion evaluation task rendered the entities more similar to the imagined ones, particularly with odd colored and odd shaped fruits.

## General Discussion

The present study aimed to investigate the role that shape and color play in the representation of natural objects. We start from an EG perspective (Barsalou, 2008; Borghi and Pecher, 2012; Scorolli, 2014), that is from the idea that cognition is grounded directly on the functioning of the nervous and bodily systems. In this framework, biofunctional comprehension would provide the basis for psychological understanding. The view we endorse does not assume knowledge as an object or the body as static; rather, underlining the fact that cognition is based on action and oriented to action, it highlights the fact that knowledge is a dynamic and highly context dependent process (Iran-Nejad, 2000, 2011). In such a framework, consider the respective role of color and shape for biofunctional understanding, which might be used as a prerequisite for psychological comprehension. Content without color is an abstract content (see Iran-Nejad and Ortony, 1984; Iran-Nejad and Bordbar, 2013): color is crucial for biofunctional understanding. Shape is more than crucial, it is intrinsically linked with biofunctional understanding. In our view objects and entities recognition requires the flexible integration of color and shape, with shape playing a more dynamic potent role than color, due to its privileged relationship with action. In sum: according to the perspective we adopt our body shapes the way we interact with objects and entities around us and the way we represent them. Moreover action potentialities might differ depending on the considered objects/entities we have to interact with and on the basis of the different situation/task we have to cope with.

We presented objects altered in color and shape and asked participants to categorize them on different dimensions. Moreover, to avoid isolating domain-specific knowledge structures from the context in which they arise and to highlight the dynamic character of knowledge structures (Iran-Nejad, 2011), we selected different kinds of tasks: to the more used semantic categorization task we added the manipulation evaluation task (i.e., a task that puts more emphasis on action) and the motion evaluation task (i.e., a task that directs attention to a different property of the considered entities: the way they move). We focused on natural objects, which are typically considered as color diagnostic, and distinguished between manipulable and not manipulable ones, i.e., between fruit and animals. As anticipated in the introduction, the importance of shape and color is compatible with both embodied and non-embodied views of cognition. However, our findings allow to disambiguate for *which tasks* shape is more/less relevant than color, as well as *why* they can play different roles.

While explicit judgments on our stimuli showed greater perceptual complexity of shape- modified entities compared to color-modified ones (regardless of the specific kind of entity), in Experiment 1 we found that shape alterations affected more strongly fruit than animals categorization. In the manipulation evaluation task (Experiment 2) we found also an effect of the kind of entity, as participants were overall faster with fruits than with animals: the manipulation evaluation task seems to implicitly require a simulation of hand interaction (Jeannerod, 1995; Johnson and Grafton, 2003). Nevertheless, forcing participants to focus on a possible manipulation, with entities that they are less used to directly handle (animals) we found that shape played a more important role than in the categorization task. Finally analyses on errors suggest that the accuracy in accomplishing the manipulation task is affected by the feasibility/unfeasibility of action: (right-handed) participants with animals performed worse for “yes in the backpack-*right hand*” responses than for “yes in backpack- *left hand* responses.” In the motion evaluation task (Experiment 3), the performance was affected only by the properties’ alterations: shape still had a more important impact than color with animals, while both color and shape alterations did not affect fruits.

Let us try to understand the experimental findings:

When we consider animate entities (animals, Predictions 1 and 3) response latencies analyses confirm our predictions that shape does have a more relevant impact than color for tasks focusing on self-propelled motion – a capability of animate entities that strongly depends on the structural arrangement of body’s parts, that is on their specific shape. Moreover the present findings extend our predictions showing that shape does have a more crucial role than color for animals also with tasks involving a possible action on the entities – which we are less used to directly handling compared to fruits. These results, highlighting the role of the specific task at hand, are in keeping with a EG views, which ascribe an important role to the relationship between body – movement – specific context and goal: for animals the alteration of some bodily parts can indeed result in impaired motion.

Consistently in the motion evaluation task shape alterations do not influence inanimate – non-self-moving entities (fruits, Predictions 1 and 2). Instead, with these manipulable entities shape seems to have a higher impact than color for the semantic categorization task. This confirms our proposal that shape is important not only for structural reasons but for its relationship with action as well. Finally with the manipulative task we still find an effect of shape (disadvantages for shape alteration condition compared to the baseline condition), consistent with the similarity between the categorization and the manipulation evaluation task in case of manipulable entities (objects are represented in terms of potential action patterns, Wilson, 2002; Barsalou, 2008; Borghi and Caruana, 2015; see also van Elk et al., 2010) but comparable to the one of color (no differences with the odd-colored condition).

The fact that the manipulation evaluation task succeeded in evoking the simulation of grasping objects is confirmed by the effect of mapping in the analysis on accuracy: responses with animals were less accurate with the right hand than with the left hand for “yes backpack” responses. This seems to suggest that manipulable and not manipulable entities differently activate the two hands.

Finally, consistently with our predictions (Predictions 1 and 4), the investigation on the long-lasting effects of the tasks (see also Scorolli et al., 2015) showed that the previous experience with a manipulation evaluation task rendered the shown entities (both animals and fruit) more similar to the imagined ones. This result is consistent with the idea that objects are represented in terms of patterns of potential action (Borghi and Riggio, 2009).

The novel used paradigm allowed us to point out that actually the odd-colored stimuli never deteriorated the performance if compared to the baseline condition, but – across tasks and entities – they differently affected the response latencies when compared to odd-shaped stimuli. Thus the present paradigm – by contrasting shape- and color- alterations – provides information on shape/color relative weight: the present findings show that shape is more crucial than color for fruit in categorization tasks and for animals in manipulation and motion tasks. Overall, clearly in keeping with EG views, our results suggest that the role of shape for animals is strictly linked to their possibility to move, and not acted upon, while the importance of shape and color for manipulable objects is strictly linked to both their possibility to be acted upon, as well as to the specific kind of action/goal of the action.

Compared to theories of object recognition that ascribe a central relevance to shape (e.g., Biederman and Bar, 1999) the novelty of this study rests on the following findings:

(a) *Shape and action.* The present research complements and extends previous findings (reviewed in the Introduction), providing evidence on response latencies, accuracy and explicit judgments. Our findings suggest that shape is important not only for structural reasons, but also for its relationship with action. But this is not the only message we can take home from our study.
(b) *Shape and flexibility.* Consistently with previous EG literature, our results show that, even if crucial, the role played by shape is modulated by the task and by the kind of stimulus. Shape is not a static property: both the nature of the processed stimuli and the actual task strongly modulate the relative weight of shape. Information on shape for animals is critical both for an unfeasible manipulation task and for a motion task; conversely with fruit information on shape is activated when prompted by a categorization task. This flexibility is compatible with an EG account and we believe it has implications for literature on affordances.
(c) *Color and flexibility.* Even if less than shape, also color matters. Looking at the recent literature, evidence shows that color is more sensitive to the contextual information provided by the task and by the objects/entities. With a Stroop task Connell and Lynnot (2009) showed not only that color is important, but that it is modulated by the linguistic context. They asked participants to read sentences and then to perform a Stroop task. They found that color naming performance was better when the ink color was typical of a given object (e.g., bear in brown ink, rather than in yellow or white ink) and when it corresponded to the color the previously read sentence implied (e.g., bear in white ink following “Joe was excited to see a bear at the North Pole”). In a similar vein, Yee et al. (2012) asked participants to perform a color-Stroop task followed by a semantic judgment task (animal or not?). Each target was preceded by primes related in color or not (e.g., emerald vs. pendant > cucumber). They found color priming effects when subjects had previously performed the color-Stroop task: the priming effect was predicted by the size of the Stroop effect. As they argue, the prominence of a conceptual characteristics can be contextually dependent, flexible and variable (for discussion see Spivey, 2007, and Kiefer and Martens, 2010).

As previously shown, studies on color recognition have typically employed naming, semantic categorization and property verification tasks. Compared to this recent literature on object recognition, the novelty of our study consists in investigating the role of color: (1) compared to shape, (2) directly altering the information provided by the visual stimuli, (3) with different color-diagnostic entities, (4) in different kinds of tasks, as to the standard categorization task we added further tasks, that put more emphasis on action or on motion. This novel paradigm has highlighted that with a categorization task odd-colored fruits differ (faster responses) from odd-shaped fruits, but odd-colored animals do not differ from odd-shaped animals; conversely with a manipulation task odd-colored fruits do not differ from odd-shaped fruits, but odd-colored animals differ (faster responses) from odd-shaped animals; finally with a motion task odd-colored animals differ (faster responses) from odd- shaped animals (while for fruits, with a task addressing the motion, both the property of color and shape do not matter at all). By showing that the property of color does have a relative different weight if compared to shape, our results confirm the flexibility of color contextual information.

Overall, our data speak in favor of a view that underlines the action-based but also the dynamic and flexible character of human knowledge organization – consistently with an EG view of cognition. These findings suggest that our understanding of the world is grounded in action, and that our body shapes the way we interact with objects and entities around us and the way we represent them; nevertheless the way knowledge on object categories is accessed is not stable/automatic but flexible and contextually modulated.

## Conflict of Interest Statement

The authors declare that the research was conducted in the absence of any commercial or financial relationships that could be construed as a potential conflict of interest.
